# Gamification, social problems, and gender in the teaching of social sciences: Representations and discourse of trainee teachers

**DOI:** 10.1371/journal.pone.0218869

**Published:** 2019-06-26

**Authors:** Delfín Ortega Sánchez, Isabel María Gómez Trigueros

**Affiliations:** 1 Department of Specific Didactics, Faculty of Education, University of Burgos, Burgos, Spain; 2 Department of General Didactics and Specific Didactics, Faculty of Education, University of Alicante, Alicante, Spain; Middlesex University, UNITED KINGDOM

## Abstract

This paper analyses the representations and discourse of 41 trainee teachers on the inclusion of gaming elements in education and their potential contribution to covering social problems in the curriculum, and to education *on* and *for* gender equality between men and women. The study follows the principles of mixed methods research, based on the transcription, coding, categorisation, and analysis of data obtained from 34 semi-structured interviews and two focus groups, in addition to their descriptive quantitative analysis. The results showed optimum reception of gamification as a useful formative strategy in the various stages of education and that it can be validly used to include gender as a category of analysis in the teaching of social sciences.

## 1. Introduction and background

The concept of gamification appeared in the early twenty-first century, and is presented with multiple definitions and from various perspectives. The term originated in the business world. With its growing popularity, usefulness, and study of its application, the term is now also used in education. Deterding [1] defines gamification as ‘the use of game design elements in non-game contexts.’ Gamification must not be confused with the simple use of play-based elements or mechanics during an activity. Its meaning goes far beyond the simple inclusion and attainment of points or levels in educational processes. Gamification is understood as the application of concepts and dynamics that are, strictly speaking, play-based, in order to stimulate the teaching–learning process and make it more attractive so that students can work on specific curriculum content [2–4]. It is about making knowledge more entertaining and motivating than it would be if it were presented in a traditional lesson [5]. Through the implementation of game mechanics, the users (students, in this case) become involved [4], key skills are developed [2], such as technological literacy, and there is greater motivation towards the content being taught [3, 6]. Another characteristic of gamification is that it allows players to develop their analytical and multitasking skills, as well as their use of creativity and imagination [7].

Putting gamified strategies into practice requires the comprehensive and systematic organisation of the teaching–learning process so that the methodology achieves its intended objectives. Point to a number of requirements for the success of a gamified educational activity [8, 9]: defining clear educational objectives; defining the attitudes, skills, and abilities to be achieved; establishing the types of students who will participate so as to adjust the objectives and activities used; detailing the game mechanics and degree of challenge, badges, and rules; and determining the tools to be used during the educational activity.

In addition to the elements cited above, some authors [10–12] consider the specific mechanisms of any play-based activity. These include the game mechanics (they allow the progress made to be visible through points or badges); dynamics (the approach must be attractive and appeal to players, and give the activity meaning as a connective thread); and aesthetics (the game’s audio-visual and technological design, which relates to the realm of emotions).

How the gamified activity is designed will, to a great extent, determine its success [13]. It is therefore essential to perform a pedagogical analysis of the play-based process, keeping in mind the functionality of the resources to be implemented.

### 1.1. Gamification and ICTs

There are no doubt attention should be given to the richness offered by the technologies as promoters of gamified learning processes [14] into both formal and non-formal educational settings. For this reason, it is important for the activities designed to be accessible from any device (tablet, mobile phone). Within the context of gamification, ICTs also provide audio-visual support; they help teachers in the teaching process and provide them with multimedia resources that capture students’ attention and interest. Furthermore, the digital format is not foreign to students because technologies form part of their more immediate social environment. When technologies are used, there is greater student involvement. It is also worth mentioning the role of ICTs as a way of automating processes to put gaming elements into practice, such as controlling the allocation of points, league tables, and changes of level.

Cobo & Moravec [15] underscore the role of technology in developing imagination, creativity, and capacity for innovation. They point out that a gamification project must use technological systems that help students think, go beyond what they know, establish relationships, and find alternative ways to achieve results. Trujillo [16] points out that gamification and ICTs change the meaning of games and the role of computers. In this way, when technologies are used within the school context, students are able to find codes they already know thanks to their social practices outside the classroom and apply them in the usual manner to the task of learning. The virtues of combining gamification and ICTs to promote greater involvement and satisfaction during learning through digital-based active and gamified methodologies is therefore emphasised.

### 1.2. Social problems, gender and gamification

There is no doubt about the educational relevance of social problems in the teaching of Social Sciences [17]. Its curricular inclusion and didactic treatment allow the formation of the critical conscience of the students, the relativization, the analysis and interpretation of facts and processes, the plural development of their personal and social identities, the respect and understanding of other identities, the defense of the social justice, and the rejection of exclusion and discrimination of any kind [18–20].

The need for social science teaching for citizen participation requires, as a consequence, the promotion of learning aimed at understanding the social reality, and the curricular inclusion of relevant social problems or socially active issues [21]. This precise inclusion of cooperative-collaborative work and planning that facilitates the critical analysis of information and the formulation of new questions in educational contexts [22]. In this sense, the mechanics and dynamics of the game can offer educational scenarios, didactic alternatives and adequate resources to achieve this goal, for the promotion of an education based on social justice and combating gender inequality [23, 24].

### 1.3. Limitations of educational gamification: Research horizons

Despite the fact that, through these methodologies, the “intention is to influence the psychological and social behaviour of the player” (p.2 [25]) some authors [13, 26, 27] warn of the lack of empirical evidence on the benefits of using gamified systems in educational contexts and, in particular, in the Social Sciences classroom [28]. This warning is based on the absence of qualitative and quantitative data that confirm behavioural changes, the increased agreement among members of teams engaged in competition, and the intensified participation or the increased motivation associated with certain activities [29, 30]. Likewise, despite the theoretical recognition of the educational advantages of gamification with technology in the didactic treatment of social problems [24], there is also not enough empirical evidence to confirm its benefits in specific classrooms of Social Sciences, or to approximate to the representations and conceptions of teachers on its potential implementation [28]. However, despite its limitations [31], from the educational sphere, there is a growing popularity of the gamified teaching proposals on gender [32].

Against this backdrop, the present study seeks, on the one hand to scrutinize the trainee teachers’ representations about the educational effectiveness of active game-based methodologies in early childhood, primary school and secondary school. While on the other hand, it seeks to explore, from the perspective of trainee teachers, the potential of these methodologies in terms of problematizing content and addressing gender inequalities in the curricula of these three stages of education. The present study is also intended to analyse the possibilities and limitations of active game-based methodologies in learning social sciences and approaching the multidimensional concept of gender as a basic category of social analysis. In this respect, the main aim of the study is to verify the possible educational contributions of active game-based methodologies in education for a democratic, plural and inclusive citizenship.

## 2. Method

### 2.1. Participants

Forty-one trainee teachers from the University of Burgos and the University of Alicante (Spain) participated in the research. Twenty-four of these were enrolled in the fourth year of the Bachelor of Primary Education, 10 in the third year of the Bachelor of Early Childhood Education, and 7 in the Master of Secondary Education. There were 36 women and 5 men, and the mean age was 23.27 years (*SD* = 2.6; range = 18 to 35).

Non-probabilistic convenience sampling was used. This was based on the researchers’ ability to access the participating sample, and their suitability with regard to the research objectives. They were selected based on a single criterion [33]: they needed to have completed and passed the teaching practice modules referred to in the respective curricula. This was seen as fundamental to the soundness and influence of their representations in practical classroom contexts.

### 2.2. Instruments

The techniques used to obtain data consisted of a semi-structured interview and a focus group. Both were used as data-collection instruments. The purpose of selecting and using the interview technique was to obtain personalised information on participants’ attitudes and representations of teaching practice. This technique was complemented by the use of two focus groups, the purpose of which was to construct group meanings and to delve into the interpretation of students’ individual narratives [34, 35].

The design of the focus questions proposed in the two techniques referred to four dimensions of analysis: concept and methodological applications of gamification (questions 1 and 2); setting of implementation (question 3); educational potential (questions 4 and 5); curriculum, gender inequalities and gamification (questions 6 and 7) data are reported in Table 1.

**Table 1:** Focus questions from the interviews and focus groups.

Q1. What do you understand by the gamification of teaching and learning processes? Could you suggest a short definition?
Q2. Do you or would you use this type of strategy in your teaching practice? If so, how?
Q3. In which educational spaces or settings do you consider the use of these strategies to be valid?
Q4. Do you believe that the gamification of social science content promotes the development of students’ thinking skills? If so, which ones? Why?
Q5. In the specific context of social science teaching, do you believe that active game-based methodologies are useful? Why?
Q6. Do you believe that the gamification of social science content would promote education *on* and *for* gender equality?
Q7. If so, which materials and which methodology do you or would you use?

### 2.3. Reliability and validity

In order to check the levels of relevance and appropriateness, the degree of internal coherence and the importance of the focus questions proposed for the research objectives, we used the Delphi method [36]. In accordance with this method, three experts in social science teaching and ICTs were selected to make their judgements on a 5-point scale about the *relevance and appropriateness*, the *internal coherence* and the *importance* of the questions to be asked, and to give their overall assessment of the construct on a scale of 1 to 10.

Stability of the results was achieved, after their consensus, following the administration of the assessment instrument in two rounds and obtaining statistically reliable results (stop criterion). To use this method, two groups were formed: a coordinating group, which was made up of the researchers from the present study and those responsible for designing the instrument, and an expert evaluating group, the characteristics of which are shown in Table 2.

**Table 2:** Professional characteristics of the group of experts.

*Professional Category*	*Area of Expertise*	*Field of Teaching and Research*
University professor	Social science teaching	Geography teaching
University associate professor	Teaching and school organisation	Educational technology
Contracted lecturer	Social science teaching	History teaching

The procedure was divided into three phases:

1. First phase (initial phase). In the first phase, the coordinating group was formed, and the problem, research objectives, and study dimensions were defined. Furthermore, a schedule was agreed upon, and the members of the group of experts were contacted.
2. Second phase (development phase). In the development phase, the draft open-ended questions to be asked with both techniques (interview and focus group) were sent electronically. We then calculated the mean and standard deviation of the overall and item-specific results obtained. Finally, we calculated Cronbach’s alpha to determine reliability in both the first and second rounds.
3. Third phase (final phase). Having reached the desired degree of consensus and achieved high reliability of the results in the second round, we terminated the procedure and sent the instrument to the three members of the group of experts.

The final results showed a mean greater than 8 in the overall assessment of the construct and narrow spread of responses for *relevance and appropriateness* (*M* = 9.1; *SD* = 0.21), *internal coherence* (*M* = 9.7; *SD* = 0.25), and *importance* (*M* = 9.9; *SD* = 0.11). The following results were also obtained: an optimum overall reliability and internal consistency value for the whole scale (*α* = 0.947); a value greater than 0.8 (*α* = 0.826) for *relevance and appropriateness*; an optimum overall value for *internal coherence* (*α* = 0.857); and an overall value of *α* = 0.804 for the *importance* of the questions asked with regard to the research objectives. In addition, the values obtained for each question on the scale were between *α* = 0.661 and *α* = 0.967 data are reported in Table 3, which was satisfactory.

**Table 3:** Descriptive statistics and reliability for each element.

	Expert group (*n* = 3) *Relevance and appropriateness*			Expert group (*n* = 3) *Internal coherence*			Expert group (*n* = 3) *Importance*		
	*M*	*SD*	*α*	*M*	*SD*	*α*	*M*	*SD*	*α*
Q1	4.60	0.36	0.847	4.76	0.40	0.967	4.60	0.36	0.854
Q2	4.10	0.52	0.709	4.26	0.25	0.814	4.26	0.25	0.755
Q3	4.43	0.37	0.746	4.43	0.37	0.794	4.43	0.37	0.701
Q4	4.60	0.52	0.736	4.76	0.25	0.830	4.60	0.52	0.671
Q5	4.23	0.64	0.737	4.40	0.36	0.807	4.23	0.64	0.661
Q6	4.96	0.05	0.857	4.63	0.55	0.768	4.30	0.51	0.820
Q7	4.93	0.11	0.867	4.60	0.52	0.767	4.93	0.11	0.854

M: average

DT: standard deviation

α: Cronbach’s alpha

To determine the validity of the seven questions, we applied a pilot test to a sample made up of more than 5% (3 students) of our research sample (41 participants). It was found that the information produced corresponded adequately to the objectives set.

### 2.4. Design and procedure

The research was conducted within the framework of cross-sectional mixed research designs, based on the application of the principles of grounded theory [37, 38] and their descriptive quantitative analysis (frequencies, percentages, modes, and standard deviations). The emerging nature of the qualitative data obtained started from the application of inductive methodology [39] and constant comparison, so as to define similarities and differences in the content analysed.

Data from the individual interviews and focus groups were collected—during an initial phase and on six different occasions—so as to obtain different perspectives on the phenomenon under investigation. During a second phase, the constant comparative method was used, with similarities and differences being identified so that the information collected could be interpreted, and categories of analysis were subsequently generated and defined until saturated [40].

The interviews were based on a semi-structured design with a total of seven focus questions, which made it possible to delimit the expected information and return richly nuanced information. Both the interviewees and the focus-group participants were required to tailor their contributions to these structural and open-ended focus questions in order to interweave topics, connect content, and construct “a holistic and comprehensive understanding of the reality” (p.337 [41]) under study.

During an initial planning phase, guiding questions were designed to generate the opinions, attitudes, and experiences of the participating students, without trying to reach consensus or make decisions regarding the subject of study in the specific context of their teaching practice.

The interviews and focus groups were organised by email and conducted in the Faculties of Education at the University of Burgos and the University of Alicante between June 2017 and May 2018. In these communications, participants were informed about the conditions in which the two techniques would be used, the objectives of the research, and its duration: 65 minutes for the individual interviews and 95 minutes for the focus groups. Each participant was also informed of the proposed composition of these groups (3 people per group).

Contributions from the researchers attempted to facilitate the participation of the trainee teachers, promoting discussion of their opinions, attitudes, and teaching narratives based on their personal experiences. These contributions were simply used when necessary to bring participants back to the question or proposed topic. At all times, the aim was to create a relaxed atmosphere in which trainee teachers’ beliefs, perceptions, and experiences could emerge as spontaneously as possible. So as to explore the reasons behind them, these beliefs, perceptions, and experiences were never questioned or debated by the moderators.

Once the purpose of the research had been explained, the participants were reminded that the data obtained would be processed and interpreted in a confidential and anonymous fashion, and that they would be audio-recorded. The notes taken by the moderators on the context in which the interviews and focus groups had been conducted were firstly organised. The information obtained was then transcribed by one of the researchers exactly as stated, avoiding textual or discursive correction.

### 2.5. Data analysis

The information yielded first underwent a careful exploratory–comparative reading of the available content, with the aim of reducing the units of analysis to significant recording units and identify thematic lines and the possible saturation of the potential categories and emerging subcategories–variables [42, 43]. Secondly, based on the constant comparative method [44], the following were conducted: an open coding process of the transcriptions, which assigned concepts to the emerging units of meaning; an axial coding process, which grouped and organised the codes obtained, based on their connections, into study variables or subcategories that explained the categories of analysis; and a selective coding process, with which the final categories, variables, or subcategories were integrated around five central dimensions of analysis. These were concept and methodological applications of gamification; setting of implementation; educational potential, gamification, and curriculum; and gamification and gender equality.

The coding of the information, its abstraction into the final categories and subcategories (variables) and their subsequent analysis followed the process of immersion–crystallisation proposed by Borkan [45]. Following the repeated and independent reading of the data by the researchers, two joint meetings were conducted in order to reach agreement on the emerging themes in each question asked (open coding). Once defined, an external researcher coded and narrowed the themes again. Finally, a third meeting was held between the two principal researchers and the external researcher in order to reach consensus on the final categories and variables for analysis (axial coding and selective coding).

Finally, when the partial existence of more than one study variable-subcategory in the statements made by the students had been identified, the statements were codified and quantified using an ordinal scale of 1 to 3, where 1 represented a low level of approximation and 3 a high level of approximation with the variable under study. This procedure made it possible to quantitatively adjust the explanatory tendencies of each emerging variable as it is observed in the Table 4.

**Table 4:** Emerging dimensions, categories, and subcategories of analysis.

	*Dimension*	Q*	*Category*	*Subcategory–variable*				Cod**
1.	Concept and methodological application	1.2	Teaching and learning strategy	Competition				v1.1
				La***	La_1_	La_2_	La_3_	
				Cooperation–collaboration				v1.2
				La***	La_1_	La_2_	La_3_	
			Teaching resource	Motivation				v1.3
				La***	La_1_	La_2_	La_3_	
2.	Setting of implementation	3	Context	Non-formal				v2.1
				La***	La_1_	La_2_	La_3_	
				Formal				v2.2
				La***	La_1_	La_2_	La_3_	
				Formal and non-formal				v2.3
				La***	La_1_	La_2_	La_3_	
3.	Educational potential	4.5	Skills	Creative thought				v3.1
				La***	La_1_	La_2_	La_3_	
				Empathy				v3.2
				La***	La_1_	La_2_	La_3_	
				Communication skills				v3.3
				La***	La_1_	La_2_	La_3_	
				Social skills				v3.4
				La***	La_1_	La_2_	La_3_	
				Technological skills				v3.5
				La***	La_1_	La_2_	La_3_	
4.	Curriculum, inequalities gender and gamification	6,7	Education *on* and *for* gender equality	Social problems				v4.1
				La***	La_1_	La_2_	La_3_	
				Gender inequalities				v4.2
				La***	La_1_	La_2_	La_3_	
			Methodology	Traditional–transmissive				v5.1
				La***	La_1_	La_2_	La_3_	
				Reproductive using ICTs				v5.2
				La***	La_1_	La_2_	La_3_	
				Active technological–digital				v5.3
				La***	La_1_	La_2_	La_3_	

Q*: connection with the question asked

*Cod*.**: coding of the variable

La***: levels of approximation

Once the patterns of content had been identified and the available information categorised, the keywords and the frequency of the most recurrent thematic responses were quantified, and the percentages of concomitant textual fragments were recorded. To present the results, the concepts of analysis were linked with the most representative responses for each category.

To identify participants, organise the recording units, and present the results, activities were coded alphabetically and students were identified alphanumerically: FG [focus group]-T_x_-D_year_ [teacher_(ID number)_-degree_(academic year)_], I [interview]- T_x_-D_year_ [teacher_(ID number)_-degree_(academic year)_].

The software ATLAS.ti (v. 7.5.4) was used to analyse the qualitative data due to its ability to code, categorise, quantify keywords, and interpret the information obtained. We used the statistical package SPSS v.24 to process and analyse the quantitative data.

## 3. Results

### 3.1. Analysis dimension 1: Concept and methodological application (questions 1 and 2)

The students used the word ‘play’ with great frequency, as well as the term ‘gamification,’ to refer to the gamification of social science content as a teaching methodology, strategy, or resource in the social science classroom, as it is observed in the Table 5.

**Table 5:** Descriptive frequencies of terms to define the concept of classroom gamification.

		*f*.
Term		
	Play	238
	Gamification	33
	Total	271
Methodology		
	Integrated methods	100
Strategy		46
Resource	Digital interactive resources	15
	ICT teaching materials	14
	Facilitatory means	41
	Video games	10
	WebQuests	5
	Internet	5
	Web	5
	ICTs	27
	Total	122

*f*. *=* frequency

The processes of educational gamification were primarily associated with instrumentalisation and resource management (*f* = 122; *f*_digital_ = 81), followed by their methodological conception (*f* = 100) from three perspectives: competition, cooperation–collaboration, and motivation (Table 6).

**Table 6:** Descriptive statistics for each study variable.

	Subcategories–variables	*n*	*f*.	%	*M*_*o*_	*SD*
1	Competition	32	5*	15.6*	3.00	0.76
			5**	15.6**		
			22***	68.8***		
	Cooperation–collaboration	82	4*	4.9*	3.00	0.47
			4**	4.9**		
			74***	90.2***		
	Motivation	150	36*	24.0*	2.00	0.74
			67**	44.7**		
			47***	31.3***		
2	Formal	27	27***	100.0***	3.00	0.00
	Formal and non-formal	82	5*	6.1*	3.00	0.55
			9**	11.0**		
			68***	82.9***		
3	Creative thought	55	5*	9.1*	3.00	0.58
			50***	90.9***		
	Empathy	96	96***	100***	3.00	0.00
	Communication skills	59	12**	20.3**	3.00	0.40
			47***	79.7***		
	Social skills	73	5**	6.8**	3.00	0.25
			68***	93.2***		
	Technological skills	64	5**	7.8**	3.00	0.27
			59***	92.2***		
4	Social problems	41	4*	9.8*	3.00	0.60
			37***	90.2***		
	Gender inequalities	96	9**	9.4**	3.00	0.29
			87***	90.6***		
	Traditional–transmissive	9	3*	33.3*	2.00	0.78
			4**	44.4**		
			2***	22.2***		
	Reproduction using ICTs	18	18***	100.0***	3.00	0.00
	Active technological–digital	18	18***	100.0***	3.00	0.00
	Total verbatim quotations	902				

*Low level of approximation

**Medium level of approximation

***High level of approximation

With the maximum level of approximation, 68.8% (*n* = 32) of the trainee teachers’ contributions defined gamification as a methodology that encourages competition between students as well as point attainment (*M*_*o*_ = 3; *SD* = 0.76):

It’s a game-related strategy. It’s a points system; that is, a competition

(I-T_2_-Primary_4_).

I regularly use it in some activities, but never all the time, nor do I turn the lesson into a competition

(I-T_6_-Secondary_1_).

I make the activities balanced in terms of points—all students can win a point

(I-T_10_-Early Childhood_3_).

Of a total of 82 verbatim quotations, 90.2% of students believe, with a high level of approximation, that educational gamification facilitates the promotion of cooperative–collaborative formative strategies (*M*_*o*_ = 3; *SD* = 0.47):

Whenever possible, I try to make use of these strategies. For example, when the students are studying kilos, half kilos, and quarter kilos, after explaining to the students what they are, I give each student a digital card with one of these terms. I tell them a specific weight such as two and a half kilos. Taking into account the value on their card, they have to form groups to get the exact weight. The students left without a group say how many kilos they need to reach the desired weight

(FG-T_11_-Primary_4_).

*They* [the students] *can play cooperative games or activities and use a form of technology*

(I-T_3_-Pre-school_3_).

There are lots of applications for cooperative work, and the whole class can participate in them with their computers

(I-T_20_-Primary_4_).

*I almost always try to make them [the activities] group ones so that they learn to work together*. *It* [gamification] *promotes cooperation in the classroom*

(FG-T_1_-Secondary_1_).

Student motivation is another of the most recognised factors. This recognition is usually connected, mainly with a medium level of approximation (*f* = 67; *n* = 150), with the need for balance between education and entertainment, the appeal of teaching resources, and students’ increased attention (*M*_*o*_ = 2; *SD* = 0.74):

Students can sometimes confuse entertainment and education. They can be interrelated concepts, but something that is educational doesn’t have to be entertaining, nor is everything that is entertaining, educational. However, in early childhood education, whenever education is entertaining, the result can be much better. You can educate in an entertaining fashion, but not 100%

(I-T_9_-Pre-school_3_).

These resources make it possible to ‘hook’ students and get more active participation

(I-T_5_-Secondary_1_)

Teaching social sciences with game-related resources gets students involved in a more personal way, with attractive challenges that capture their attention

(I-T_22_-Primary_4_).

These perceptions are followed, with the highest level of approximation, by considerations about the advantages of gamification in increasing the motivation of students and getting them to become truly involved in their own learning process

(*f =* 47; *n* = 150):

Gamification is an active methodology that uses the motivating principles of games to achieve pedagogical objectives

(I-T_7_-Secondary_1_).

It’s an educational method that tries to involve and motivate students, which is different to traditional teaching methods. Games are not only used in academic contexts, but also in social ones, because they put students in a range of situations, which doesn’t happen in traditional contexts

(I-T_17_-Primary_4_).

The term ‘gamification’ is aimed at working on theoretical content in a more practical fashion, so it’s more motivating for students

(FG-T_2_-Pre-school_3_).

### 3.2. Analysis dimension 2: Setting of implementation (question 3)

Eighty-two point nine percent (*n* = 82) of the trainee teachers’ responses appreciate, with a high level of approximation, the teaching potential of gamification strategies in both formal and non-formal educational contexts (*M*_*o*_ = 3; *SD* = 0.55). On the other hand, with a frequency of 27 quotations, some students use them in formal contexts only (*M*_*o*_ = 3; *SD* = 0.00):

I consider them valid for both educational spaces because, on a teaching level, the objectives are the same as those of other active methodologies

(I-T_6_-Secondary_2_).

I appreciate their potential in every setting. In early childhood education, any space can be turned into an educational setting

(I-T_6_-Pre-school_3_).

The strategies are more appropriate for formal spaces. In reality, we’re aiming to work on the classroom curriculum in an active, practical, and motivating way

(I-T_12_-Primary_4_).

### 3.3. Analysis dimension 3: Educational potential (questions 4 and 5)

The trainee teachers specified, at different levels of approximation, the educational potential of the processes of gamification, which can be divided into five factors: empathy (*n* = 96), development of creative thought, and integrated communication (*n* = 59), social (*n* = 73) and technological (*n* = 64) skills.

With a total frequency of 96 verbatim quotations, the students appreciated the methodological capacity of educational gamification to develop students’ personal, social, and historical empathy, by understanding the perspectives and contexts in which social information is created (*M*_*o*_ = 3; *SD* = 0.00):

For example, if we’re working on a historical event such as colonisation in the classroom, we can design a play with scenes. In addition to putting ourselves in the shoes of historical figures and being able to imagine what they felt or why events took place as they did, the students are participants in their own learning, so the content will be absorbed more permanently

(FG-T_7_-Primary_4_).

Through traditional or digital games, students can put themselves in the shoes of historical figures and find out about the social context in which they lived. With traditional teaching, it’s difficult to develop historical empathy. We can make students feel like they’re in the skin or context that we’re teaching them about

(I-T_14_-Primary_4_).

The gamification of learning processes helps students put themselves in the shoes of people from the periods being studied, see how they may have felt, whether they’d have acted in that way

(I-T_2_-Secondary_1_).

With a frequency of 50 interventions (*n* = 55), trainee teachers, with the maximum level of approximation, pointed to the educational potential of gamification to develop students’ creative thought (*M*_*o*_ = 3; *SD* = 0.58):

With these strategies, various methods could be used for problem-solving

(I-T_13_-Primary_4_).

Everything that’s attractive for students will foster the development of creativity and imagination

(I-T_5_-Pre-school_3_).

Game-based methodologies bring them closer to the problems of the past or the present and allow them to reflect on possible solutions

(I-T_7_-Secondary_1_).

Along these lines, the educational use of games appears to contribute to the development of communication skills (*f =* 47, *n* = 59) through the interaction of students to solve mutual challenges (*M*_*o*_ = 3; *SD* = 0.40); social skills (*f =* 68, *n* = 73) through conflict resolution, consensus, and group decisions (*M*_*o*_ = 3; *SD* = 0.25); and technological skills (*f =* 59, *n* = 64) through handling and technical mastery of digital devices (*M*_*o*_ = 3; *SD* = 0.27):

It will help students to have bolder views and make critical judgements

(I-T_4_-Secondary_1_).

Social and communication skills are closely related to group games. Likewise, gamification is currently difficult to understand without a connection to ICTs

(I-T_7_-Secondary_1_).

Students almost always have to play in groups and with the rest of the class. To do so, the use of social and communication skills is essential. The same applies to technological skills. A lot of gamification is done using a computer

(FG-T_6_-Primary_1_).

Through the game, students exchange opinions, emotions, feelings, and establish connections. These are often cooperative digital games, and these types of games promote empathy and social relations

(I-T_8_-Early Childhood_3_).

*They allow for group work and thus the development of social skills*. *Through games*, *they* [students] *can develop technological skills because they use technologies to interact*, *and therefore to acquire social skills in the development of content*

(I-T_21_-Primary_4_).

Every time games are incorporated into the classroom or any context, they’re working on social and communication skills, because with the word ‘game’ comes the implicit idea of relationships, communication, socialisation… Also technology, because gamification of the classroom has progressed considerably with the incorporation of new technologies

(FG-T_18_-Primary_4_).

There are many digital applications for cooperative work. The whole class can participate with their computers

(I-T_15_-Primary_4_).

### 3.4. Analysis dimension 4: Curriculum, inequalities gender and gamification (questions 6 and 7)

In the dataset, the dimension *curriculum*, *inequalities gender and gamification* received one of the highest frequencies and absolute percentages for its two variables. These indicators also recorded maximum levels of approximation in the explanation of the subcategories of analysis *social problems* (*f* = 41, *n* = 37; *M*_*o*_ = 3; *SD* = 0.60) and *gender inequalities* (*f* = 96, *n* = 87; *M*_*o*_ = 3; *SD* = 0.29).

The trainee teachers expressed their complete agreement with the ability of gamified social content to facilitate coverage of contemporary social problems (particularly inequalities) in the curriculum, and of gender as a category of social analysis:

Games lead to students tackling situations or problems themselves, which they have to learn to solve by themselves. These could be minor problematic situations or major social problems

(I-T_7_-Primary_4_).

The best way to work on social problems is to recreate these situations in the classroom and get students to develop critical thinking towards inequalities or certain social realities

(I-T_4_-Early Education_3_).

They’re useful for addressing social problems from the past or present such as gender inequality. Personally, I’ve addressed it during an innovative activity

(FG-T_24_-Primary_4_).

Children reproduce gender roles and stereotypes, so games could be an ideal resource to try to eliminate these prejudices

(I-T_22_-Primary_4_).

For example, role plays allow students to put themselves in the shoes of women from antiquity and see how they were treated, to imagine how they would feel if they acted in that way, etc. That is, they bring students closer to inequalities between men and women, which, until only recently, were very marked

(FG-T_23_-Primary_4_).

With gamification, the history of gender can be visualised, giving a voice to forgotten women. Many women continue to be invisible in traditional historical narratives

(I-T_3_-Secondary_1_).

From this dimension, the trainee teachers also focused their discourse on the methodological possibilities offered by gamification for education on gender equality. Their contributions were classified based on three methodologies: traditional–transmissive (*M*_*o*_ = 2; *SD* = 0.78), reproductive using ICTs (*M*_*o*_ = 3; *SD* = 0.00), and active technological–digital (*M*_*o*_ = 3; *SD* = 0.00).

The first of the methodologies had mainly medium levels of approximation with the study variable. It included gamified ICT resources with no real methodological consequences for education on gender equality (*f* = 4, *n* = 9):

Personally, I think there are topics that should first be tackled using a traditional form of education. Once certain concepts have been absorbed, gamification could be a great developmental methodology for the treatment of the gender perspective, in parallel with the teacher’s explanations

(FG-T_6_-Secondary_1_).

The reproductive methodology using ICTs follows the defining principles of the first approach, but it incorporates technological resources as additional supports for the traditional methodology (*f =* 18, *n* = 18):

Education for gender equality could be introduced in debates and meetings, interactive stories, projecting images and adapted videos, asking questions about them…

(I-T_1_-Primary_4_).

From the third perspective, education *on* and *for* gender equality should use active methodologies that incorporate digital teaching strategies, methodologies, and resources in an integrated and operational manner (*f =* 18, *n* = 18):

Such as using WebQuests, where students have to analyse the latest information on gender inequalities

(I-T_10_-Primary_4_).

With games or web pages designed for teaching purposes, and oriented towards investigation so that students can learn the true role played by women, not only throughout history, but in life in general

(I-T_5_-Secondary_1_).

## 4. Discussion and conclusions

Motivation is one of the most acclaimed potentialities of gamified methodologies [46–49, 12], which was confirmed in the present study. Valderrama [50] indicates that teaching approaches that use games trigger intrinsic motivation through the simple pleasure of playing, doing away with the mandatory nature of traditional activities, which are driven by extrinsic motivation [51], in educational contexts. The implementation of some elements unique to games, such as levels, points, and badges increases the amount of time participants spend on the activity and the likelihood that they will continue to participate. However, González et al. [52] assert that game-based approaches in the classroom may produce the opposite of the intended objective. Indeed, it has been shown that gamified activities increase competitiveness, discouragement when challenges are not overcome, abandonment of activities, and lack of agreement among participants [53].

According to the opinion of some authors, the belief that gamification is successful is essentially based on the idea that games are “entertaining” [54], which was one of the expressions frequently collected in this study. This ignores the fact that, ultimately, they are a mechanism to increase effort. Such a position, confirmed by the participants in this study, discredits the motivating value of the gamified activities themselves, and portrays them as a kind of ‘trap’ for the participants, who are unaware of the real purpose of the proposed gamified activity.

In the same way, educational gamification is also valuable in *immersive* formative experiences [55], which are formative actions that aim to delve into and acquire broader knowledge of the content being studied through the entertainment brought about by the game. Gamification therefore reinforces learning, with improved educational performance in the educational contexts in which it is implemented [56], with students showing greater dedication. From this perspective, this methodology enriches project-based learning as it actively involves students and promotes collaborative learning [57], teamwork, individual and collective decision-making, as well as collaborative attitudes and behaviours [56], and promotes the development of dynamics focused on modifying certain negative behaviours, which generates healthy social habits [58]. This methodological approach, related to the resolution of challenges, is confirmed in the statements of the students participating in this research. Accordingly, a sense of healthy competition to obtain group or individual points, tolerance of errors as a natural part of learning, curiosity and experimental learning through simulation of the activity or project appear to facilitate a shift away from apathy towards the knowledge being taught. However, despite the fact that the trainee teachers referred to the motivating potential of games, they also described competition and the attainment of points as a risky direction in which formal gamified spaces may be moving.

From a constructivist perspective, another educational possibility offered by gamification is the gradual construction of learning by the students when they interact with novel, rich, and diverse environments [59]. To this can be added the development of compromise and autonomy [60, 61] through overcoming challenges and skill acquisition, and superior thinking and coordination skills [62]. Activities gamified with technology also promote the stimulation of certain affective factors such as the development of student interaction and social skills. In this respect, the participating students recognise, in the gamification of historical–social content, the development of three types of skills (communication, social, and technological), and of other cognitive processes (empathy and creative thinking during problem-solving) [63–65].

The educational potential of play-based simulation or gamified contexts [66] and their connection with the real world have served as a starting point for a few studies and proposals on the use of games (and videogames) in educational settings [3, 51, 55, 67, 68, 69]. From this viewpoint, the connection between action and learning [66], and problem-solving [3] is confirmed in the representations of trainee teachers when they refer to tackling social problems in the curriculum with gamified strategies and resources.

The use of game mechanics and dynamics, directed towards educating students on the equality of men and women, is a new way to reach an audience that knows the message, but needs to internalise it. Teaching actions confirm that these strategies are able to strengthen the positive attitudes of participants, training students to think critically about gender-based stereotypes. The use of gamification in formative contexts does therefore appear to promote actions aimed at reducing certain social problems such as gender inequality, generating changes and educating future citizens. Indeed, the results show that optimum reception of games and the processes of gamification are useful strategies in the teaching–learning of social science content throughout the various stages of education and in problematizing this content; moreover, they can be validly used to include gender as a category of analysis in the teaching of social sciences, a field, so far, unexplored in the training contexts of teachers of compulsory education. The diagnostic nature of these results seeks to encourage teachers who are making curriculum decisions to implement specific teaching actions generally geared towards the use of gamification in the teaching of social problems, and, in particular, of gender inequality.
